# Characteristics and associated factors of physical activity-related injuries among university students in southern China

**DOI:** 10.1038/s41598-020-61197-7

**Published:** 2020-03-06

**Authors:** Weicong Cai, Dongchun Tang, Yang Gao, Wenda Yang, Shangmin Chen, Lijie Gao, Cunxian Jia, Liping Li

**Affiliations:** 1 Injury Prevention Research Center, Shantou University Medical College, Shantou, Guangdong China; 2 Department of Non-communicable Disease Control and Prevention, Shenzhen Center for Chronic Disease Control, Shenzhen, Guangdong China; 3 Department of Sport and Physical Education, Hong Kong Baptist University, Kowloon Tong, Hong Kong China; 4 Department of Epidemiology, Shandong University School of Public Health, Jinan, Shandong China

**Keywords:** Diseases, Risk factors

## Abstract

This cross-sectional study aimed to describe the characteristics of physical activity-related injury (PARI) and to explore its associated factors among university students in Chaoshan district. Selected from the baseline survey in March and April, 434 students graded 1–3 from two universities were interviewed face-to-face in April and May 2017. Socio-demographics, physical activity (PA) participation, risk-taking behaviors, and PARI occurrences in the past 12 months were collected. Group Lasso logistic regression was applied to identify the risk factors of PARI. Totally, 317 PARI episodes were reported by 184 subjects with an overall injury risk of 0.73 injuries/student/year (males: 1.00, females: 0.63) and an injury incidence density of 0.81 injuries per 1000 PA exposure hours (males: 1.13, females: 0.69). Most injuries involved the lower extremities and were sprains and strains. Males, sports team members, and those with high-risk rebellious and anti-social behaviors were more likely to sustain PARI compared to their counterparts. Those who participated in vigorous-intensity PA with longer duration (particularly ≥ 150 min/week) were at a higher risk for PARI. This study indicates that PARI is a health concern among university students and great efforts should be taken to prevent them from PARI when promoting a physically active lifestyle.

## Introduction

The World Health Organization (WHO) has recommended that adults aged 18–64 years should take part in at least 150 minutes of moderate-intensity physical activity (MPA), or 75 minutes of vigorous-intensity PA (VPA), or an equivalent combination of moderate- to vigorous-intensity PA (MVPA) throughout the week^1^. However, a recent report from the WHO global health observatory data repository showed that 31.1% of the adults were physically inactive^2^, which was considered as the fourth leading risk factor for the increasing prevalence of non-communicable diseases, accounting for more than 3 million preventable deaths globally^3^. Being physically active benefits our physical, cognitive, and psychological well-being individually^4,5^. In light of these advantages of PA participation, almost all countries and regions have involved in the current global PA promotion. Of them, several countries have even published PA guidelines involving frequency and duration to various populations^6–8^.

PA promotion is public health priority at present, while a potentially rising risk of physical activity-related injury (PARI) may be unavoidable underlying the worldwide emphasis on a physically active lifestyle^9^. In fact, PARI has a negative effect on PA participation, which has been demonstrated in different age groups, genders, and PA levels^9–11^. Previous reports on PARI incidence among active populations varied from 1.91 to 8.44 per 1000 athlete-exposures^10–12^. In addition to directly substantial socioeconomic burden, PARI can lead to indirectly adverse consequences like physical and psychological discomfort and social implications^13,14^. Worse still, in the long term, a history of injury is recognized as an essential risk factor for PARI predisposition^15^ and increases the risk of other health problems like osteoarthritis^16^. These disadvantages run counter to the initial aim of PA promotion. Thus, taking effective and successful injury-prevention measures have great potential public health gains.

According to the “Sequence of prevention” model, in order to develop preventive strategies, descriptive epidemiology should be conducted to describe the characteristics and etiology of PARI^17^. To date, most injury studies have mainly focused on children and adolescents and collegiate athletes^9–11^, which revealed that gender, age, year level, body mass index (BMI), PA level, and family environment were related to the occurrence of PARI^18–21^. However, evidence about the epidemiological study on PARI specifically for general university students is scarce. Based on our previous baseline investigation^22^, we carried out this study to further verify the PARI occurrence and collect the details of each PARI episode among university students. Hence, the aims of this study were to describe the characteristics of PARI and to explore the potential associated factors contributing to PARI occurrence by the method of group Lasso (i.e., least absolute shrinkage and selection operator) logistic regression among university students in southern China.

## Material and methods

### Study participants

A two-stage study was conducted in two universities (one comprehensive university and one normal university) in Chaoshan district, southern China. In the first stage, 2123 students in 1st, 2nd, and 3rd year were recruited into the baseline survey by the method of cluster random sampling in March and April 2017. In the second stage, nearly one-fifth of the selected students (n = 434, 20.4%) consented and completed the face-to-face interviews in April and May 2017. There were no significant differences in distribution of the basic demographics (i.e., gender, age, and year level) between the study subjects in two stages (*P*-values range from 0.07 to 0.18). The purpose of the study and the instruction of the questionnaire were verbally explained to study participants, and the informed consent was sent prior to the interviews.

This study was conducted according to the Declaration of Helsinki and approved by the Shantou University Medical College Ethics Committee (SUMC-2016-22).

### Data collection

Socio-demographics of the participants included gender, age, year level, screen time (including cellphone and computer usage), and sports team membership. We measured height and weight, waist and hip circumferences on the sites, which were calculated into BMI and waist-hip ratio (WHR), respectively. In addition, ages, socioeconomic status (job and education level) and marital status of students’ parents were also collected.

PA participation on a weekly basis in the past 12 months was evaluated via a series of standardized questions adapted from the short version of the Minnesota Leisure Time Physical Activity Questionnaire (MLTAQ)^23^ and the Children’s Leisure Activities Study Survey Chinese version (CLASS-C)^24^. It possesses sound reliability in this study (Cronbach’s α = 0.795). A total of 31 moderate-intensity or vigorous-intensity physical activities during school physical education (PE) classes, sports, transportation, and leisure time in a typical week were collected. Students were inquired whether they participated in any intensities of PA such as basketball, football, tennis, badminton, tennis, and volleyball weekly during the past 12-month periods. Those with a positive response were further required to provide information on the frequency (total cumulative times) and duration (average minutes each time) of this type of PA on both a weekday and a weekend, respectively, and then the weekly MPA and VPA participation (total cumulative minutes per week) were calculated. According to the WHO’s recommendations related to PA participation for adults^1^, students were grouped into different categories (MPA:<150, 150 to <300, and ≥300 min/week; VPA: <75, 75 to <150, and ≥150 min/week, respectively) based on their average weekly participation in various intensities of PA. The overall self-reported PA participation (i.e., VPA combined with MPA, hours/year) in the past 12 months was then estimated.

Risky behaviors of students were evaluated using the revised Chinese version of the Risk-taking Questionnaire-Risk Behavior Scale, which was validated to possess good reliability (Cronbach’s α = 0.766) and has been confirmed to have good one-week test-retest reliability in earlier studies^25,26^. A total of 17 items in this scale were divided into four factors including thrill-seeking (five items, i.e., snow skiing, taekwondo fighting, inline skating, parachuting, and entering a competition), rebellious (six items, i.e., leaving school, underage drinking, smoking, getting drunk, staying out late, and drinking and driving), reckless (two items, i.e., taking drugs and having protected sex), and anti-social risks (four items, i.e., overeating, teasing and picking on people, cheating, and talking to strangers). For each item, students were asked to endorse one of five responses: 0 (would never do), 1 (would hardly ever do), 2 (would do sometimes), 3 (would do often), and 4 (would do very often). By summing up the score for each risk-taking behavior factor, participants were classified into two different groups (i.e., high-risk and low-risk) according to their scores of being higher or lower than the median.

PARI is any injury resulting from during periods of PA participation including PE classes, sports activities, transportation, or leisure-time PA. A countable PARI episode must occur during the past 12-month periods and meet one or more of four judgment criteria, which was fully described in previous studies^18,19^: the student (a) has to stop the current PA immediately and/or (b) cannot participate in the next planned PA and/or (c) is absent from class the next day and/or; (d) has to seek medical attention (i.e., from providers ranging from first aid personnel to general physicians or physiotherapists). The further details of each self-reported PARI episode including time, place, cause, type, injured body part, severity, and activity in which the injury occurred were described by the injured students. The detailed information enabled us to validate the measure of outcome PARI.

To test the reliability of the information collected by face-to-face interviews, the interviewed data were validated and reliability-tested against the data taken by telephone survey among 50 students with a time interval of one-week after their completion of the earlier interviews (average kappa coefficient = 0.775 ± 0.262).

### Statistical analysis

Injury risk (IR) was calculated as the total amount of injuries per number of students during the past 12-month periods, and injury incidence density (IID) was calculated as the total number of injuries per 1000 PA exposure hours^10^. The 95% confidence intervals (CIs) of the IIDs were calculated based on a Poisson distribution^27^. Categorical variables were expressed as number and percentage and continuous variables were presented as mean and standard deviation (SD) or median and interquartile range (IQR). Pearson chi-square tests (or Fisher’s exact tests) and independent-sample *t* tests (or non-parametric tests) were used to analyze the group-between differences in study variables. The multivariable logistic regression model was performed to explore the independent factors associated with PARI, where odds ratios (ORs) and 95% CIs of significant variables were calculated. All significant study variables were selected in a forward manner (likelihood ratio) with the selection criteria of α_in_ = 0.05 and α_out_ = 0.10. SPSS 23.0 (SPSS Inc. Chicago, IL, USA) was used for statistical analyses. All statistical tests were two-sided, and a *P*-value less than 0.05 was considered significant.

Recently, penalized likelihood-based methods have received much attention. The group-wise Lasso variable selection operator, selecting the whole study factors instead of the individual dummy variables, was used to explore the potential risk factors of diseases^28^. We thereby applied a group Lasso logistic regression model to identify risk factors of PARI occurrence in the current study, which was established by the ‘grpreg’ package within version R 3.4.2^29^.

## Results

### General information of participants

Overall, 434 university students (319 females and 115 males) in 1st, 2nd, and 3rd year were included in our interviews, with a mean age of 20.03 years (SD = 1.21). Males and sports team members experienced a higher portion of PARI than their counterparts (both *P* < 0.05). In addition, VPA participation with various duration would significantly differ in PARI occurrence (*χ*^2^ = 8.610, *P* = 0.014) (Table 1).

**Table 1:** Comparison of main variables investigated of participants with physical activity-related injury or not.

Characteristics	All (N = 434)	Non-PARI (N = 250)	PARI (N = 184)	*P*-value
Gender				0.042
Female	319 (73.5)	193 (60.5)	126 (39.5)	
Male	115 (26.5)	57 (49.7)	58 (50.3)	
Study year				0.596
Year 1	187 (43.1)	104 (55.6)	83 (44.4)	
Year 2	145 (33.4)	83 (57.2)	62 (42.8)	
Year 3	102 (23.5)	63 (61.8)	39 (38.2)	
Age, years, mean (SD)	20.03 (1.21)	20.06 (1.26)	19.98 (1.15)	0.432
Sports team member				0.001
No	290 (66.8)	183 (63.1)	107 (36.9)	
Yes	144 (33.2)	67 (46.5)	77 (53.5)	
BMI (kg/m^2^)				0.444
<18.5	109 (25.1)	68 (62.4)	41 (37.6)	
18.5 to 23.9	286 (65.9)	163 (57.0)	123 (43.0)	
24.0 to 27.9	27 (6.2)	14 (51.9)	13 (48.1)	
≥28.0	12 (2.8)	5 (41.7)	7 (58.3)	
Screen time				0.239
<5 h/d	103 (23.7)	52 (50.5)	51 (49.5)	
5 to <10 h/d	249 (57.4)	148 (59.4)	101 (40.6)	
≥10 h/d	82 (18.9)	50 (61.0)	32 (39.0)	
VPA participation				0.014
<75 min/week	123 (28.3)	78 (63.4)	45 (36.6)	
75 to < 150 min/week	72 (16.6)	49 (68.1)	23 (31.9)	
≥150 min/week	239 (55.1)	123 (51.5)	116 (48.5)	
MPA participation				0.320
<150 min/week	5 (1.2)	4 (80.0)	1 (20.0)	
150 to <300 min/week	61 (14.1)	31 (50.8)	30 (49.2)	
≥300 min/week	368 (84.8)	215 (58.4)	153 (41.6)	
Warming-up				0.694
Never	7 (1.6)	4 (57.1)	3 (42.9)	
Occasionally	168 (38.7)	101 (60.1)	67 (39.9)	
Often	162 (37.3)	94 (58.0)	68 (42.0)	
Always	97 (22.4)	51 (52.6)	46 (47.4)	
Suitable clothes/shoes				0.113
Never	26 (6.0)	18 (69.2)	8 (30.8)	
Occasionally	122 (28.1)	79 (64.8)	43 (35.2)	
Often	151 (34.8)	81 (53.6)	70 (46.4)	
Always	135 (31.1)	72 (53.3)	63 (46.7)	
Mother’s educational levels				0.284
Primary school or below	177 (40.8)	98 (55.4)	79 (44.6)	
Middle school	169 (38.9)	96 (56.8)	73 (43.2)	
High school	53 (12.2)	37 (69.8)	16 (30.2)	
Vocational school or above	35 (8.1)	19 (54.3)	16 (45.7)	
Father’s educational levels				0.716
Primary school or below	79 (18.2)	42 (53.2)	37 (46.8)	
Middle school	196 (45.2)	116 (59.2)	80 (40.8)	
High school	109 (25.1)	61 (56.0)	48 (44.0)	
Vocational school or above	50 (11.5)	31 (62.0)	19 (38.0)	

PARI, physical activity-related injury; SD, standard deviation; BMI, body mass index, was calculated by weight (kg)/height^2^ (m); VPA, vigorous-intensity physical activity; MPA, moderate-intensity physical activity; PA, physical activity.

^a^*P*-value, the group-between differences were determined by independent-sample *t* tests and Pearson’s *χ*^2^ tests or Fisher’s exact tests.

^b^Figures in parentheses indicate percentages.

A total of 317 PARI episodes (females: 202, males: 115) were reported by 184 participants (females: 138, males: 56), leading to an overall IR of 0.73 injuries/student/year (males: 1.00; females: 0.63). Medians and IQRs for MPA and VPA participation (min/week) were 632 (390–990) and 180 (60–328), respectively. The weekly participation in MPA and VPA were 797.14 (SD: 617.86) and 249.01 (SD: 179.96) minutes on average, respectively, resulting in a mean duration of PA participation of 1046.15 minutes per week (i.e., 17.44 h/week; females: 17.58, males, 17.03, *P* = 0.352). This equals an overall IID of 0.81 injuries per 1000 PA exposure hours (95% CI: 0.73–0.90). There was a significant difference in IID between males (1.13, 95% CI: 0.94–1.35) and females (0.69, 95% CI: 0.60–0.79).

### Main characteristics of participants with PARI

As shown in Table 2, a total of 398 injured body parts occurred in 317 PARI episodes. Approximately two-thirds (66.1%, n = 263) of injuries located at the lower extremities—particularly ankle and knee, 16.6% (n = 66) sustained to the upper extremities, and the same percent (5.8%, n = 23) occurred to trunk, head, and neck, and was heatstroke, respectively. There was a comparable difference in different genders (*χ*^2^ = 12.544, *P* = 0.250), and male students had a markedly higher percentage of injuries to the lower extremities compared to females (70.8% vs. 63.6%).

**Table 2:** Distribution of injured body parts among injured participants.

	Total	Males	Females
Lower extremities			
Ankle/foot/toe	127 (31.9)	47 (34.3)	80 (30.7)
Knee/shin/calf	80 (20.1)	32 (23.4)	48 (18.4)
Thigh	44 (11.1)	16 (11.7)	28 (10.7)
Hip	12 (3.0)	2 (1.5)	10 (3.8)
Sub-total	263 (66.1)	97 (70.8)	166 (63.6)
Upper extremities			
Wrist/hand/finger	34 (8.5)	15 (10.9)	19 (7.3)
Shoulder/upper arm	15 (3.8)	6 (4.4)	9 (3.4)
Elbow/forearm	17 (4.3)	2 (1.5)	15 (5.7)
Sub-total	66 (16.6)	23 (16.7)	43 (16.5)
Trunk			
Upper/lower back	13 (3.3)	4 (2.9)	9 (3.4)
Chest/abdomen	10 (2.5)	2 (1.5)	8 (3.1)
Sub-total	23 (5.8)	6 (4.4)	17 (6.5)
Head/face/neck	23 (5.8)	7 (5.1)	16 (6.1)
Heat stroke	23 (5.8)	4 (2.9)	19 (7.3)
Total	398 (100.0)	137 (34.4)	261 (65.6)

^a^Figures in parentheses indicate percentages.

Overall, 381 injuries were reported by the injured students. Of which, sprains (n = 149, 39.1%), strains (n = 82, 21.5%), and laceration or abrasion (n = 55, 14.4%) were the top three frequently injured types, but non-statistically significant difference was found between males and females (*χ*^2^ = 13.937, *P* = 0.083) (Table 3).

**Table 3:** Type of the injury among injured participants.

Characteristics	Total	Males	Females
Sprains	149 (39.1)	58 (43.3)	91 (36.8)
Strains	82 (21.5)	35 (26.1)	47 (19.0)
Contusion	19 (5.0)	8 (6.0)	11 (4.5)
Dislocation/fracture	5 (1.3)	2 (1.5)	3 (1.2)
Tendinitis	8 (2.1)	3 (2.2)	5 (2.0)
Laceration/abrasion	55 (14.4)	15 (11.2)	40 (16.2)
Sunstroke	23 (6.0)	3 (2.2)	20 (8.1)
Sunburn	18 (4.7)	3 (2.2)	15 (6.1)
Others	22 (5.8)	7 (4.5)	15 (6.1)
Total	381 (100.0)	134 (35.2)	247 (62.8)

^a^Figures in parentheses indicate percentages.

### Risk factors associated with PARI occurrence

**Figure 1:**
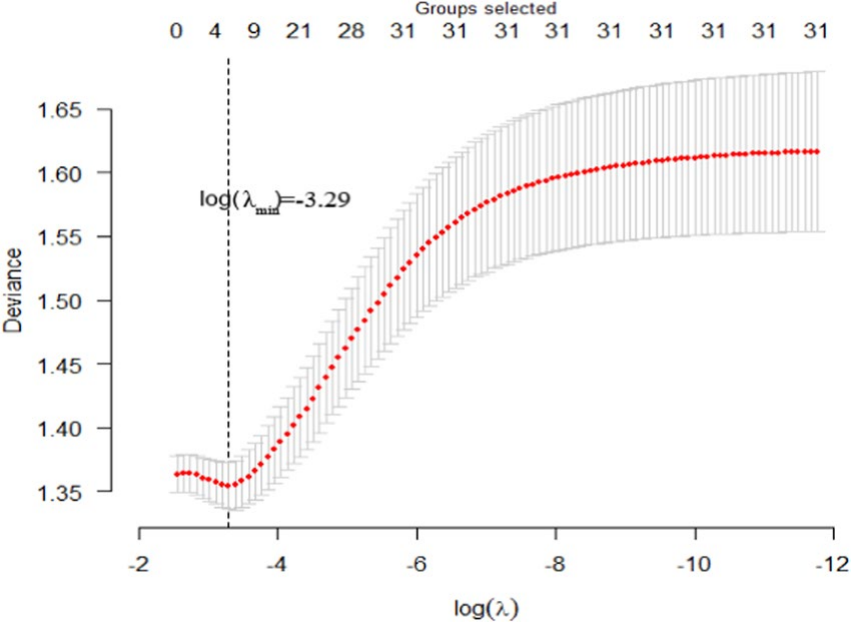
Ten-fold cross-validation for the deviance with error bars of the group Lasso logistic regression model across various values of the tuning parameter λ (log-scale). The total number of covariates is 31 and the corresponding number of dummy variables is 53. The minimum cross-validated deviance reached 1.35 when the log of λ is −3.29, which is the corresponding optimal model. R software 3.4.2 was used to produce this image.

**Figure 2:**
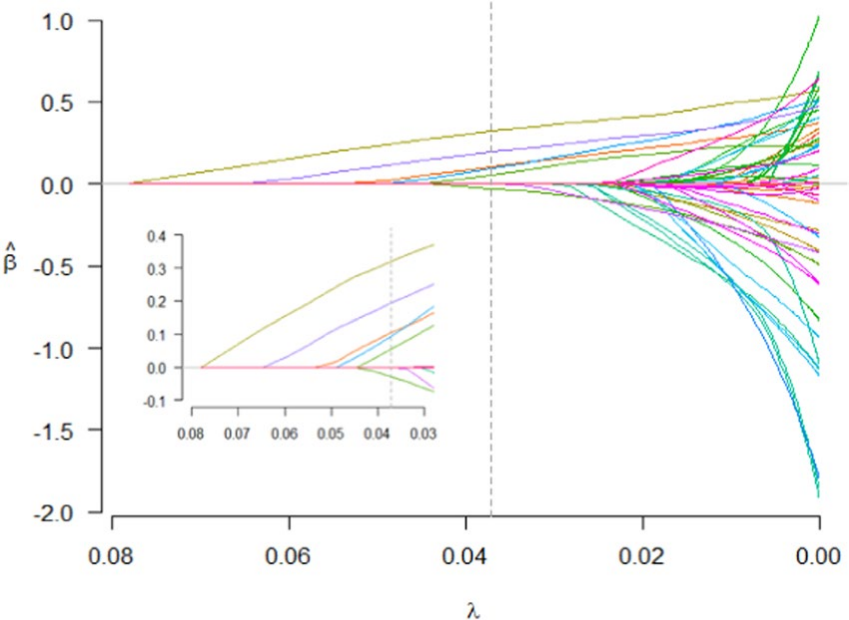
The path of the coefficients estimated over a grid of values for λ. The numbers of selected covariates were five and one for the two tuning parameter λ. A group of covariates including sports team members, anti-social behavior, gender, rebellious behavior, VPA participation of at least 150 min/week, and VPA participation of 75 to <150 min/week from top to bottom corresponding to λ = 0.0371 were selected. R software 3.4.2 was used to produce this image.

Figure 1 presents the cross-validation deviance and the numbers of selected variables at a grid value of λ (log-scale) for the group Lasso logistic regression model. Based on the results of 10-fold cross-validation, five groups of statistically significant covariates were identified corresponding to the minimum cross-validated deviance when the log of λ was −3.29. The corresponding regularization paths for the fitted group Lasso regression models across different values of λ are displayed in Figure 2. Five covariates including sports team members, anti-social behavior, gender, rebellious behavior, and VPA participation (two tuning parameter λ) were finally selected when the value of λ was 0.0371 with the optimal deviance of 1.35.

A multivariate logistic regression model with all significant covariates was performed as a comparison, whose results are summarized in Table 4. Totally, six factors were selected by the general logistic regression model. Since there were more categorical covariates in this study, it was more appropriate to select the whole factor in the model using a group-wise selection like group Lasso logistic regression model. As showed in two models, male students and sports team members were more vulnerable to sustain PARI (Lasso’*β* = 0.104, OR = 1.583; Lasso’*β* = 0.320, OR = 1.880, respectively). Participation in VPA with longer duration (especially ≥ 150 min/week) would increase the risk for PARI occurrence (Lasso’*β* = 0.056, OR = 1.484). Moreover, students with high anti-social and/or rebellious risk were also more likely to suffer from PARI (Lasso’*β* = 0.095, OR = 1.533; Lasso’*β* = 0.193, OR = 1.653, respectively).

**Table 4:** Comparison of the estimates of PARI-related factors from the multivariate logistic regression model and the group Lasso logistic regression model.

Variables	Lasso’s *β*	*P*-value	Logistic’s *β*	OR (95% CI)
Gender	0.104			
Female				1.000 (ref.)
Male		0.045	0.459	1.583 (1.002–2.501)
Sports team member	0.320			
No				1.000 (ref.)
Yes		0.003	0.631	1.880 (1.248–2.834)
VPA participation	0.056			
<75 min/week				1.000 (ref.)
75 to <150 min/week		0.523	−0.204	0.815 (0.436–1.525)
≥150 min/week		0.042	0.595	1.484 (1.150–2.646)
Rebellious behavior	0.095			
Low risk				1.000 (ref.)
High risk		0.047	0.427	1.533 (1.001–2.934)
Anti-social behavior	0.193			
Low risk				1.000 (ref.)
High risk		0.017	0.503	1.653 (1.092–2.502)
Father’s age				
<50 years old				1.000 (ref.)
≥50 years old		0.046	−0.711	0.692 (0.459–0.997)

OR, odds ratio; CI, confidence interval; VPA, vigorous-intensity physical activity.

**Figure 3:**
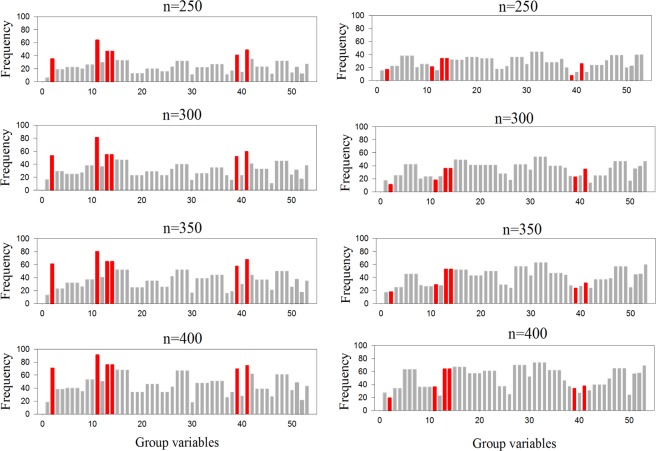
Group Lasso with λ = −3.29 (log-scale) on the simulated data. In each scenario, the frequency output of the bootstrapped data is on the left, whereas the frequency output of the permuted data is on the right. The red bars stand for gender, sports team members, VPA participation of at least 150 min/week, VPA participation of 75 to <150 min/week, rebellious and antisocial behavior in turn. R software 3.4.2 was used to produce this image.

We applied the group coordinate descent algorithm to the 100 sets of simulated data with various sample sizes (n = 250, 300, 350, and 400). Figure 3 shows the validation results of the group Lasso logistic regression model on both bootstrapped and permuted data. Five significant covariates were selected most all the time in the bootstrapped data, while the random and unpredictable selection of covariates in the permuted data indicated that the previously significant associations among the response and the predictors disappeared. Therefore, the results of the group Lasso logistic regression method were demonstrated to be robust in this study.

## Discussion

This study provided an up-to-date description of the incidence and characteristics of PARI among general university students in southern China. Among the whole subjects, 317 PARI episodes were totally self-reported by 184 injured students during the past 12 months. The overall IR was 0.73 injuries/students/year in general university students, which was slightly lower than the previous report of 0.85 found in physical education teacher education (PETE) students^10^. Similarly, both studies indicated that male students had a higher risk of PARI experience than females (1.00 vs. 0.63 and 0.89 vs. 0.78, respectively). In addition, our findings revealed an overall IID of 0.81 injuries per 1000 PA exposure hours with a greater IID in males compared to female counterparts (1.13 vs. 0.69). Irrespectively of gender, however, the IIDs were clearly lower than those observed in other studies. For example, the IIDs among National Collegiate Athletic Association (NCAA) male and female volleyball players were 4.69 and 7.07 per 1000 athlete-exposures, respectively^30^, while NCAA football players had even higher IIDs of 8.44 and 8.07 in males and females, respectively^12^. One possible explanation for this discrepancy is the differences in PA levels. The higher PA level was accompanied by the increase of injury occurrence, and this increase might be more obvious in collegiate athletes^31^. Other factors like study design and injury definition may also contribute to the divergent results^10,31^. Although the IR and IID among general university students were obviously lower than those of sports-active populations, we still should pay enough attention to this problem especially when promoting PA for the public.

In this study, we found that nearly three-fifths (60.6%) of injuries were sprains and strains, which was in line with previous reports from different populations^11,12,31^. Additionally, lower extremity was the most frequently injured body part, accounting for 66.1% of all injuries. Regardless of sports-active population, this result was aligned with those reported in earlier research^12^. For example, about 71.5% of NCAA male and female football injuries occurred to the lower extremities^12^. Strikingly, the higher percentage (74.3%) of lower extremity injuries was reported by Goossens *et al*. in PETE students^10^. Disparities in the injury location might be due to the following possibilities. Firstly, this might be owing to anatomical and neuromuscular differences in the lower extremities, and/or discrepancies in PA levels^32^. Other factors like the frequent involvement in the majority of PAs, constant acceleration or deceleration, and potentially highly loaded frontal plane movements might also contribute to the high rates of lower extremity injuries^11^. These particular injury characteristics and the potential contributors to lower extremity injuries should be taken into account, which is of great help to develop appropriate and targeted prophylactic injury-intervention to reduce the occurrence of PARI across the whole body.

In order to adapt future injury prevention measures to the population-specific characteristics of general university students, we explored the common associated factors for PARI by the method of group Lasso logistic regression. Similar to previous studies^19,31,33^, we found that male students had a higher risk of sustaining PARI compared to females. Some researchers ascribed this partly to the higher PA participation of males^10^. This could not be supported by the average PA exposure time in the present study. Nonetheless, we speculated that the higher VPA volume might play a more important role in PARI occurrence (males: 345.46 min/week; females: 215.06 min/week). Also, males were more likely to take part in more competitive team sports like basketball and football^19^. The high possibility of contact, jumping, sprinting, and/or pivoting activity acting as the major injury mechanisms are commonly found in these PAs^33^. In addition, sports team members had a nearly two-fold greater likelihood to suffer from PARI than their counterparts. This might be related to the higher PA levels they involved, and put them at higher risk of exposure to PARI^19^. Therefore, future specific prevention strategies should be implemented to reduce the occurrence of PARI among these high-risk populations.

Physically active lifestyle is helpful to achieve and maintain individual health at an optimal level^4,5^. Our study revealed that the majority of university students were highly active according to the WHO’s recommendations on PA participation for adults. The following reasons may contribute to this trend. First, university students in China might be more physically active due to their release from heavy academic pressure for college admission and their independence from parents^34,35^. Besides that, in the past decade, the Chinese government has made great efforts on PA promotion^36^. Our findings may reflect its positive impact on students in universities. In addition, students might over-report their PA levels out of social desires^37^. Nevertheless, students with higher VPA levels would elevate their exposure to PARI. Our study showed that VPA participation with longer duration would significantly increase the susceptibility to PARI, particularly those with VPA duration of at least 150 min/week. This was highly in parallel with the previous results^38^, noting that high intensity and long duration of PA participation were the primary injury contributors (a supportive explanation for males were more likely to suffer from PARI as well). However, neither the WHO’s PA recommendations nor other countries’ PA guidelines provide any suggestions on frequency, duration, and intensity from a safety view^1,6,7^. Clearly, there was a knowledge gap in safe PA participation (especially VPA), which needs further research.

Risk-taking behaviors have been identified as a major determinant of injury^39^. The findings of our study revealed that students with high-risk rebellious and/or anti-social behaviors had a higher risk for PARI (OR = 1.533 and 1.653, respectively). Previous studies noted that cognitive performance might be in relation to risk-taking behaviors, which led individuals to distort the potential risk of a specific behavior^40^. The impulsiveness and adventurousness inherent in individuals might also act a part^33^. This was aligned with the result of our earlier study—poor individual safety awareness could increase the risk of PARI occurrence^19^. Optimization of environments might help the reduction of injuries when taking part in risk-taking behaviors, which will serve as a protective strategy in terms of injury^39^. Thus, there is an urgent call for theory-based multifaceted actions that focus on high-risk populations to reduce individual risk behaviors and to enhance the quality of sports environments, aiming at reducing the PARI in relation to risk-taking behaviors when we attach great importance to a physically active lifestyle.

In this study, we applied the group Lasso logistic regression method to identify the most important associated factors contributing to the increased risk of PARI. As we knew, a structured questionnaire always contains numerous variables, it is thus a complex prediction model that might not be suitable to explore the inner characteristics due to the problem of multicollinearity. Both the consistent results of traditional general logistic regression and group Lasso logistic regression method and its stable simulation verified by bootstrap and permutation tests in the present study indicated that it was feasible to solve that problem. However, some limitations should also be taken into consideration. The data of PA participation was self-reported, which might be over-reported by the study subjects out of socially desirable. Although the results of one-week test-retest were validated to be reliable, the measure of PA would be more accurate if objectively measuring tools were used and its dynamic changes especially considering the negative effect of PARI occurrence would be able to capture at the same time. Besides that, we have applied several ways to ensure the validation of outcome PARI measure, but we still could not fully preclude the possibility that some participants might not report their PARI occurrence accurately due to their memory. This might have an influence on the estimation of IR and IID. In addition, selected from the first stage of the baseline survey, the participants in this study were not a completely random sample, which may affect the generalizability of our findings to some extent.

In summary, PARI is a health problem among university students in southern China. The majority of injuries occurred in the lower extremities, and sprains and strains were the primary types of injury. We identified that males, sports team members, those with longer duration of VPA participation, and those with high-risk rebellious and/or anti-social behaviors were more vulnerable to suffer from PARI. Hence, coordinated and arduous efforts are required to maximize the benefits of PA and minimize the risks of PARI.
